# Adult Neurogenesis in the Female Mouse Hypothalamus: Estradiol and High-Fat Diet Alter the Generation of Newborn Neurons Expressing Estrogen Receptor α

**DOI:** 10.1523/ENEURO.0027-16.2016

**Published:** 2016-09-22

**Authors:** Elizabeth P. Bless, Jane Yang, Kalpana D. Acharya, Sabin A. Nettles, Fair M. Vassoler, Elizabeth M. Byrnes, Marc J. Tetel

**Affiliations:** 1 Neuroscience Program, Wellesley College, Wellesley, Massachusetts 02481; 2 Department of Biomedical Sciences, Cummings School of Veterinary Medicine, Tufts University, Grafton, Massachusetts 01536

**Keywords:** estrogens, leptin, obesity, pSTAT3

## Abstract

Estrogens and leptins act in the hypothalamus to maintain reproduction and energy homeostasis. Neurogenesis in the adult mammalian hypothalamus has been implicated in the regulation of energy homeostasis. Recently, high-fat diet (HFD) and estradiol (E2) have been shown to alter cell proliferation and the number of newborn leptin-responsive neurons in the hypothalamus of adult female mice. The current study tested the hypothesis that new cells expressing estrogen receptor α (ERα) are generated in the arcuate nucleus (ARC) and the ventromedial nucleus of the hypothalamus (VMH) of the adult female mouse, hypothalamic regions that are critical in energy homeostasis. Adult mice were ovariectomized and implanted with capsules containing E2 or oil. Within each hormone group, mice were fed an HFD or standard chow for 6 weeks and treated with BrdU to label new cells. Newborn cells that respond to estrogens were identified in the ARC and VMH, of which a subpopulation was leptin sensitive, indicating that the subpopulation consists of neurons. Moreover, there was an interaction between diet and hormone with an effect on the number of these newborn ERα-expressing neurons that respond to leptin. Regardless of hormone treatment, HFD increased the number of ERα-expressing cells in the ARC and VMH. E2 decreased hypothalamic fibroblast growth factor 10 (*Fgf10*) gene expression in HFD mice, suggesting a role for Fgf10 in E2 effects on neurogenesis. These findings of newly created estrogen-responsive neurons in the adult brain provide a novel mechanism by which estrogens can act in the hypothalamus to regulate energy homeostasis in females.

## Visual Abstract

**Figure:**
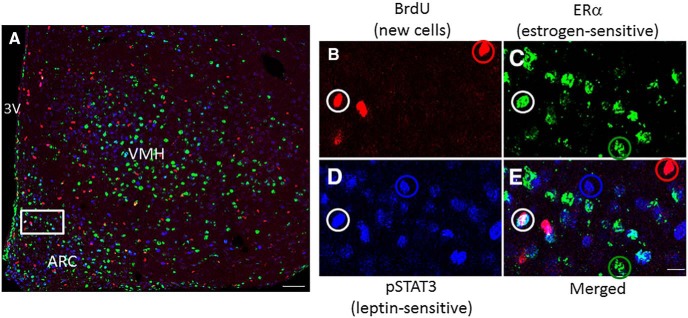

## Significance Statement

Estrogens and leptin act in the hypothalamus to profoundly impact energy homeostasis in humans and rodents. For example, postmenopausal women gain weight, increasing their risk for heart disease and diabetes. Hypothalamic neurogenesis has been implicated in energy homeostasis in adult male and female rodents. In the present study, newborn neurons that respond to estrogens and leptins were identified in the adult female mouse hypothalamus. Moreover, the generation of these newborn hypothalamic neurons was regulated by estradiol and a high-fat diet (HFD). Estradiol decreased hypothalamic *Fgf10* gene expression in mice consuming an HFD, suggesting a role for Fgf10 in estradiol effects on neurogenesis. These findings provide a novel mechanism by which estrogens can act in the female hypothalamus to regulate energy homeostasis.

## Introduction

The peripheral maintenance of energy homeostasis in mammals is profoundly influenced by hormone signaling. In particular, estrogens affect energy homeostasis through the regulation of adiposity (Heine et al., 2000; Naaz et al., 2002), activity (Wade, 1972; Blaustein and Wade, 1976), and thermogenesis (Hosono et al., 1997; Opas et al., 2006). For example, postmenopausal women gain fat weight, which increases their risk for heart disease and type 2 diabetes (Guo et al., 1999; Carr, 2003). In support of these anorectic effects of estrogens, ovariectomized rodents demonstrate a decrease in activity, and an increase in feeding and weight gain (Wade, 1972; Xu et al., 2011). Investigations of the central effects of estrogens on energy homeostasis have focused on neuropeptide expression in hypothalamic areas (Frank et al., 2014). For example, estradiol (E2) decreases mRNA expression of orexigenic factors [e.g., neuropeptide Y (NPY) and Agouti-related protein; Pelletier et al., 2007; Silva et al., 2010] and increases mRNA expression of anorexigenic factors [e.g., pro-opiomelanocortin (POMC) in the arcuate nucleus (ARC) of ovariectomized mice and rats; Pelletier et al., 2007]. While estrogen receptor (ER) α, ERβ, and membrane-associated ER have been implicated in estrogen effects on energy homeostasis (Qiu et al., 2006; Frank et al., 2014), ERα appears to contribute more to energy homeostasis (Heine et al., 2000; Santollo et al., 2007, 2010; Frank et al., 2014). Importantly, ERα function in the hypothalamus is critical in weight maintenance (Musatov et al., 2007; Xu et al., 2011).

Leptin, secreted by adipose tissue, acts in the hypothalamus to play a critical role in energy homeostasis (Sahu, 2003; Varela and Horvath, 2012; Rosenbaum and Leibel, 2014; Park and Ahima, 2015). There is cross talk between the estrogen and leptin signaling pathways in the regulation of weight, adiposity, and energy intake and expenditure (Gao and Horvath, 2008; Clegg, 2012; Nestor et al., 2014). Leptin effects on energy homeostasis are enhanced by estrogens (Ainslie et al., 2001; Clegg et al., 2006). In support, leptin receptors are colocalized with ERs in the ARC of the rat (Diano et al., 1998). It has been suggested that the signaling of these two hormones overlap via phosphorylation of the downstream signaling molecule, signal transducer and activator of transcription-3 (STAT3; Gao and Horvath, 2008).

Much is known about the generation of new neurons in the olfactory bulb and the dentate gyrus of the adult hippocampus (Zhao et al., 2008). A variety of factors influences cell proliferation and neurogenesis in the hippocampus, including estrogens (Galea et al., 2006; Pawluski et al., 2009; Duarte-Guterman et al., 2015). In addition, neurogenesis has also been observed in the adult mammalian hypothalamus, and recent work has focused on the origin and function of these new cells (Lee et al., 2012; Haan et al., 2013; Rojczyk-Gołębiewska et al., 2014). New cells in the hypothalamus are affected by diet (Lee et al., 2012; Li et al., 2012; McNay et al., 2012; Gouaze et al., 2013; Bless et al., 2014), and help to regulate energy homeostasis in male mice (Kokoeva et al., 2005; Pierce and Xu, 2010; Lee et al., 2012; Gouaze et al., 2013; Li et al., 2014) and female mice (Lee et al., 2014). In addition, high-fat diet (HFD) and E2 alter cell proliferation and neurogenesis in the ARC and ventromedial nucleus of the hypothalamus (VMH) of adult female mice (Bless et al., 2014). Furthermore, HFD and E2 affect the number of newborn leptin-responsive [phosphorylated STAT3 (pSTAT3)-expressing] neurons in the female hypothalamus (Bless et al., 2014).

The current study tested the hypothesis that the adult female mouse brain is capable of generating new ERα-expressing cells in the ARC and VMH. In addition, we asked whether newly generated ERα cells were also leptin sensitive. Last, a variety of growth factors, cytokines, and apoptotic factors influence adult neurogenesis in the mammalian hippocampus, olfactory bulb, and hypothalamus (Endres et al., 1998; Fink et al., 1998; Pencea et al., 2001; Kokoeva et al., 2005; Sasaki et al., 2006; Zhang et al., 2006; Yuan, 2008; Zhao et al., 2008; Choi et al., 2009; Li et al., 2012). The expression of some of these factors was examined in the hypothalamus for their potential role in increasing cell proliferation in obese female mice consuming an HFD.

## Materials and Methods

### Animals

For the immunohistochemistry experiments, a set of brain sections from a previously published study (Bless et al., 2014) was used. Briefly, C57BL/6 female mice (10–12 weeks of age) from the breeding colony were housed two per cage and maintained on a 12 h light/dark cycle. Mice were bilaterally ovariectomized and implanted subcutaneously with a silastic capsule (Ingberg et al., 2012) containing either 50 µg of 17β-E2 dissolved in 25 µl of 5% ETOH/sesame oil (Rissman et al., 2002; Kudwa et al., 2009) or vehicle (Veh; 5% ETOH/sesame oil). Three days after surgery, mice were either started on an HFD containing 58% kcal from fat in the form of lard (35.2% fat, 36.1% carbohydrate, and 20.4% protein by weight; catalog #03584, Harlan Teklad) or maintained on standard rodent chow (STND) containing 13.5% kcal from fat (catalog #5001, Purina).

Mice were randomly assigned to one of the following four treatment groups: STND-Veh (*n* = 5); STND-E2 (*n* = 6); HFD-Veh (*n* = 9); and HFD-E2 (*n* = 8). Six animals that did not receive the complete BrdU infusion due to the loss of the cannula during the experiment (*n* = 4) or the cannula missing the lateral ventricle (*n* = 2) were excluded from the analysis. Mice were weighed every 5 d, and the amount of food eaten was recorded every other day (1–2 h before lights off) throughout the study. Seven days after ovariectomy/silastic capsule implantation, mice were implanted with a cannula aimed at the right lateral ventricle [anteroposterior (AP), 0.3 mm; ML, 1.0 mm from bregma; DV, 2.5 mm; Paxinos and Franklin, 2004]. The cannula was attached to an Alzet osmotic pump (0.5 µl/h, 7 d; catalog #1007D, Durect) filled with 100 µl of home-made artificial CSF containing 1µg/µl BrdU (Sigma-Aldrich) and 1 µg/µl mouse serum albumin (Sigma-Aldrich) via a catheter. Mice were maintained on their respective diets for 34 d after the start of BrdU infusion to allow newborn cells to become functionally mature (van Praag et al., 2002; Kim et al., 2014b). Thirty-four days after the start of BrdU infusion, mice were deprived of food overnight. On the next day, cardiac perfusion with 4% paraformaldehyde was performed 45 min after an injection of leptin (5 mg/kg, i.p.; Peprotech). Leptin was administered in order to induce phosphorylation of STAT3 in the hypothalamus (Frontini et al., 2008). Following perfusion, brains were dissected out, postfixed in 4% paraformaldehyde for 2 h and then transferred to 20% sucrose/0.1 m phosphate buffer for 2 d until sectioning. Thirty-five-micrometer-thick brain sections were cut on a freezing microtome and stored in cryoprotectant at −20°C until processing.

### Gene expression analysis by reverse transcription-quantitative PCR

For the reverse transcription-quantitative PCR (RT-qPCR) studies, C57BL/6 female mice (8–10 weeks) from Wellesley College Animal Facility were housed on a 12 h light/dark cycle. The mice were bilaterally ovariectomized and implanted with a silastic capsule containing either 50 µg of E2 dissolved in 25 µl of 5% ETOH/sesame oil (*n* = 7) or Veh (5% ETOH/sesame oil; *n* = 7). On day 11 after ovariectomy and capsule implantation, mice were started on an HFD, as described above. All animal procedures were approved by the Institutional Animal Care and Use Committee and were conducted in accordance with the National Institutes of Health *Guide for the Care and Use of Laboratory Animals*.

### Triple-label immunohistochemistry

Sections from the anterior, medial, and posterior ARC (based on mouse brain atlas of Paxinos and Franklin, 2004; AP: −1.46, −2.06, and −2.54, respectively) and VMH (AP: −1.22, −1.7, and −2.06, respectively) were selected for analysis. Brain sections were rinsed in 0.05 m Tris-buffered saline (TBS), incubated in TBS containing 0.01 m glycine for 30 min, rinsed, and then incubated in TBS containing 0.05% sodium borohydride for 20 min to reduce autofluorescence as a result of aldehyde fixation. DNA was denatured for BrdU detection by incubating tissue in 2N HCl at 40°C for 40 min followed by rinses in borate buffer, pH 8.5, and TBS. Nonspecific antigen-binding sites were blocked with TBS containing 0.4% Triton X, 10% normal serum (donkey and goat; Lampire Biological), and 1% hydrogen peroxide for 30 min. Sections were incubated overnight at 4°C in a primary antibody cocktail containing rat anti-BrdU [dilution, 1:400; OBT0030G (RRID:AB_609567), Accurate] and rabbit anti-ERα directed against the N-terminal amino acids 21–32 of human ERα [dilution, 1:100; AB3575 **(**RRID:AB_303921), Abcam]. Tissue was washed in TBS followed by incubation with goat anti-rabbit Fab fragment (30 µg/ml; 111-006-003 (RRID:AB_2337920), Jackson ImmunoResearch Laboratories) for 2 h at room temperature. This step was necessary because two antibodies raised in the same host species (rabbit) were used. The goat anti-rabbit Fab fragment was used to block the rabbit antigen sites so that another antibody produced in rabbit (anti-pSTAT3) could be used without cross signaling (https://www.jacksonimmuno.com/technical/products/protocols/double-labeling-same-species-primary). Sections were rinsed with TBS and incubated in a cocktail of fluorescently labeled secondary antibodies including donkey anti-rat (dilution, 1:200; Cy3, Jackson ImmunoResearch Laboratories), and donkey anti-goat (dilution, 1:200; Alexa Fluor 488, Life Technologies) for 2 h at room temperature followed by rinses in TBS. Tissue was then incubated in rabbit anti-pSTAT3 [dilution 1:50; catalog #9145 (RRID:AB_2491009), Cell Signaling Technology] overnight at 4°C. The pSTAT3 antibody recognizes STAT3 only when it is phosphorylated at tyrosine 705. As shown on Western blot, this antibody binds to a specific band at ∼79 kDa in hypothalamic tissue from mice injected with leptin (Bless et al., 2014). Tissue was then rinsed in TBS and incubated in donkey anti-rabbit (dilution, 1:200; Alexa Fluor 647, Life Technologies), followed by washes in TBS. Sections were mounted on SuperFrost Plus slides (Fisher), coverslips were applied with Fluorogel (Electron Microscopy Sciences), and slides were stored at 4°C until confocal analysis.

A variety of controls were performed to confirm the specificity of this triple-label technique. To ensure that there was no cross-labeling between the two rabbit antibodies (ERα and pSTAT3), the same protocol described above was conducted with the omission of the primary antibody for either ERα or pSTAT3. There was no cellular labeling detected with the donkey anti-rabbit Alexa Fluor 647 or donkey anti-goat Alexa Fluor 488, respectively, indicating that there was no cross-labeling by either of these secondary antibodies for the inappropriate primary antibody, thus resulting in no false labeling of ERα- or pSTAT3-containing cells.

### Confocal microscopy

Images of the left hemisphere (contralateral to BrdU administration) of each brain region were taken at 400× with a TCS SP5 II Confocal Microscope (Leica Microsystems) equipped with an argon laser 488, a helium-neon laser 543, a helium-neon laser 633, and a motorized stage. In total, the following six regions of interest (ROIs) were imaged according to Paxinos and Franklin (2004): three ROIs representing the anterior, medial, and posterior levels of the ARC; and three ROIs representing the anterior, medial, and posterior levels of the VMH. Gain and offset settings were optimized for each fluorescent label separately for each ROI, and these settings were kept constant for all images within an ROI. A stack of 10 sections (1 μm each) was taken through the *z*-plane of each ROI.

### Image analysis

Images were analyzed using the Nikon NIS Elements Advanced Research software (version 3.22; RRID:SCR_002776). The size and position of each ROI was kept constant across images. One section per ROI was examined, and the total area analyzed for each ROI was as follows: ARC: 83,362, 57,170, and 128,323 μm^2^, respectively, for anterior, medial, and posterior; and VMH: 90,071, 156,401, and 122,316 μm^2^, respectively, for anterior, medial, and posterior.

Images resulting from the excitation of each of the three laser channels were merged to create a single RGB image. Each image was calibrated using a scale bar of 100 µm. A threshold intensity to optimize the signal-to-noise ratio for each RGB channel was set and kept constant across all images of an ROI. Size (minimum–maximum, 5–70) and circularity (0.35–1) restrictions were applied to distinguish cells from background. In each ROI, the number of immunoreactive cells (clustered pixels above threshold) and the average pixel intensity of each cell were collected for BrdU, ERα, and pSTAT3.

Brain sections from the same animals used in a previous study (Bless et al., 2014) that had been immunolabeled for the neuronal marker Hu, pSTAT3, and BrdU were analyzed to determine the percentage of pSTAT3-labeled cells that also expressed Hu, and thus were neurons. Three mice from each experimental group were randomly selected for the analysis. Thirty pSTAT3-labeled cells of varying intensities were randomly chosen from the medial ARC and VMH to examine the coexpression of Hu in these cells.

### TaqMan RT-qPCR

#### Brain dissection

Thirty-five days after the start of the HFD, mice were killed via CO_2_ inhalation. Mice were decapitated; brains were removed; and, under RNAse-free conditions, hypothalami were extracted and immediately frozen on dry ice until processing. For gene expression analysis, hypothalamic tissue was sonicated in lysis/binding buffer, and total RNA was extracted using the mirVana miRNA Isolation Kit (Life Technologies), as outlined in the manufacturer protocol. RNA concentration was measured with a NanoDrop (Thermo Scientific). cDNA was synthesized using the QuantiTect Reverse Transcription Kit (Qiagen).

#### RT-qPCR

RT-qPCR was performed using an AB 7500 Real-Time PCR System (Applied Biosystems) under the following standard amplification conditions: 2 min at 50°C, 10 min at 95°C, and 40 cycles of 15 s at 95 followed by 1 min at 60°C, as outlined in the manufacturer protocol. TaqMan Gene Expression Assays (Applied Biosystems) were used as PCR primers. The Gene symbol and Assay ID for the primers used were as follows: Rps16 (Mm01617542_g1); CNTF (Mm04213924_s1); Bdnf (Mm04230607_s1); Fgf10 (Mm00433275_m1); Ikbkb (Mm0122247_m1); Bcl2 (Mm00477631_m1); and Casp3 (Mm01195085_m1).

### Quantification of gene expression

Quantification of gene expression was based on the comparative cycle threshold (ΔCT). Each target was run in either duplicate or triplicate, and the raw CT values were averaged. The housekeeping gene Rps16 was used as an endogenous control to which each target gene was normalized (Meadows and Byrnes, 2015). Rps16 was verified as a suitable housekeeping gene as the CT values did not differ between treatment groups. Next, the ΔCT for each target gene was normalized against the highest expressing ΔCT value in order to obtain the ΔΔCT.

### Statistical analysis

A two-way ANOVA (diet and hormone) was run for each brain area separately. Where there were significant effects, a Tukey’s HSD *post hoc* test was used for comparisons between groups. For analysis of the RT-qPCR experiments, data were analyzed with *t* tests. SPSS, version 21 (IBM) was used for all statistical analyses. Differences were considered statistically significant at *p* < 0.05.

## Results

### Weight and food intake

As described above in the Materials and Methods section, the tissue analyzed in the present study was collected from a previous cohort of animals (Bless et al., 2014). As previously reported (Bless et al., 2014), animals treated with E2 ate less than animals treated with Veh, and HFD-Veh animals weighed ∼35% more than animals in all other treatment groups.

### The adult female hypothalamus generates new ERα-expressing cells

Consistent with previous findings (Bless et al., 2014), the current study revealed an interaction between hormone and diet with an effect on the number of new cells throughout the female mouse ARC (Table 1; *p* = 0.022^a^ for anterior regions, *p* = 0.002^b^ for medial regions, and *p* = 0.001^c^ for posterior regions) and VMH (*p* = 0.002^d^ for anterior regions, *p* = 0.003^e^ for medial regions, *p* = 0.003^f^, and posterior regions). Mice consuming an HFD had increased cell proliferation that was attenuated by E2 in the medial ARC (*p* = 0.026^g^), posterior ARC (*p* = 0.021^h^), and medial VMH (*p* = 0.001^i^).

**Table 1:** Statistics

	Data structure	Type of test	*p* Values
a	Normally distributed	ANOVA	0.022
b	Normally distributed	ANOVA	0.002
c	Normally distributed	ANOVA	0.001
d	Normally distributed	ANOVA	0.002
e	Normally distributed	ANOVA	0.003
f	Normally distributed	ANOVA	0.003
g	Normally distributed	Tukey’s HSD *post hoc*	0.026
h	Normally distributed	Tukey’s HSD *post hoc*	0.021
i	Normally distributed	Tukey’s HSD *post hoc*	0.001
j	Normally distributed	ANOVA	0.02
k	Normally distributed	ANOVA	0.019
l	Normally distributed	ANOVA	0.044
m	Normally distributed	Tukey’s HSD *post hoc*	0.11
n	Normally distributed	Tukey’s HSD *post hoc*	0.15
o	Normally distributed	Tukey’s HSD *post hoc*	0.15
p	Normally distributed	Tukey’s HSD *post hoc*	0.11
q	Normally distributed	ANOVA	0.023
r	Normally distributed	ANOVA	0.014
s	Normally distributed	ANOVA	0.04
t	Normally distributed	Tukey’s HSD *post hoc*	0.152
u	Normally distributed	Tukey’s HSD *post hoc*	0.14
v	Normally distributed	Tukey’s HSD *post hoc*	0.145
w	Normally distributed	Tukey’s HSD *post hoc*	0.096
x	Normally distributed	ANOVA	0.043
y	Normally distributed	ANOVA	0.054
z	Normally distributed	ANOVA	0.048
aa	Normally distributed	ANOVA	<0.001
bb	Normally distributed	Tukey’s HSD *post hoc*	0.014
cc	Normally distributed	Tukey’s HSD *post hoc*	0.002
dd	Normally distributed	Tukey’s HSD *post hoc*	<0.001
ee	Normally distributed	Tukey’s HSD *post hoc*	0.02
ff	Normally distributed	Tukey’s HSD *post hoc*	<0.001
gg	Normally distributed	Student’s *t* test	0.027

**Figure 1.**
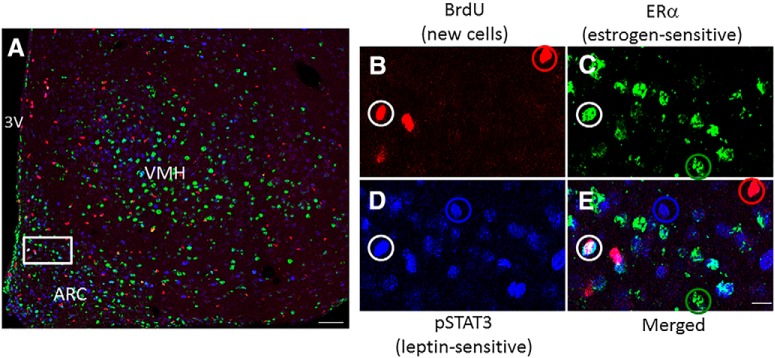
The adult female mouse ARC and VMH generate newborn neurons that respond to estrogens and leptin. ***A***, Photomicrograph of the medial ARC and VMH from a Veh-treated mouse fed an HFD at 400× magnification shows ERα (green), BrdU (red), and pSTAT3 (blue) labeling. ***B–E***, A magnified view of the outlined area in the ARC from ***A*** shows BrdU cells (red circle; ***B***), ERα cells (green circle; ***C***), pSTAT3 cells (blue circle; ***D***), and a triple-labeled neuron (white circle; ***E***). 3V, Third ventricle. Scale bars: ***A***, 50 μm; ***B–E***, 10 μm.

Cells double labeled with BrdU and ERα were found in all regions examined (Fig. 1; see Fig. 3*A*,*B* ). As with cell proliferation, there was an interaction between hormone and diet in the number of new ERα-expressing cells. The interaction was seen in the medial ARC (*F*_(1,27)_ = 6.19, *p* = 0.02^j^), and in the anterior (*F*_(1,26)_ = 6.37, *p* = 0.019^k^) and medial (*F*_(1,25)_ = 4.54, *p* = 0.044^l^) VMH. Although further analysis revealed no significant differences between groups, there was a pattern for HFD to increase the number of new ERα-expressing cells in the medial ARC (*p* = 0.11^m^, STND-Veh vs HFD-Veh) and the medial VMH (*p* = 0.15^n^, STND-Veh vs HFD-Veh), and for E2 to attenuate this effect (*p* = 0.15^°^ for medial ARC; and *p* = 0.11^p^ for medial VMH; HFD-Veh vs HFD-E2). This pattern is consistent with the effects of HFD and E2 on cell proliferation, as described above.

### A subpopulation of new ERα neurons responds to leptin

**Figure 2.**
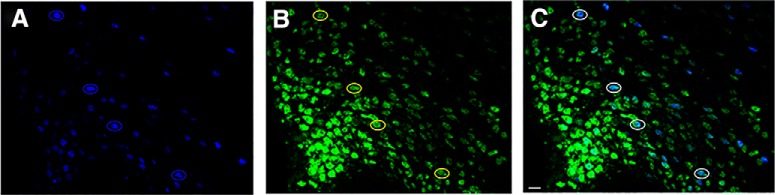
Virtually all pSTAT3-immunopositive cells in the medial ARC express a neuronal marker, Hu. ***A–C***, Representative image of the medial ARC from a Veh-treated animal consuming an STND shows pSTAT3 cells (blue circle; ***A***), Hu cells (yellow circle; ***B***), and double-labeled neurons (white circle; ***C***). Magnification, 400×. **S**cale bar, 10 µm.

Images from a previous study (Bless et al., 2014) of medial ARC and VMH sections were analyzed to determine the percentage of pSTAT3-labeled cells that express the neuronal marker Hu. Of the 30 randomly chosen pSTAT3-immunopositive cells in the medial ARC per animal, 99.4 ± 0.4% also expressed Hu, indicating that these pSTAT3 cells are neurons (Fig. 2). Similarly, in the medial VMH, 98.5 ± 0.7% of pSTAT3 cells coexpressed the neuronal marker Hu. These findings indicate that virtually all pSTAT3-labeled cells in the ARC and VMH of the present study are neurons.

**Figure 3.**
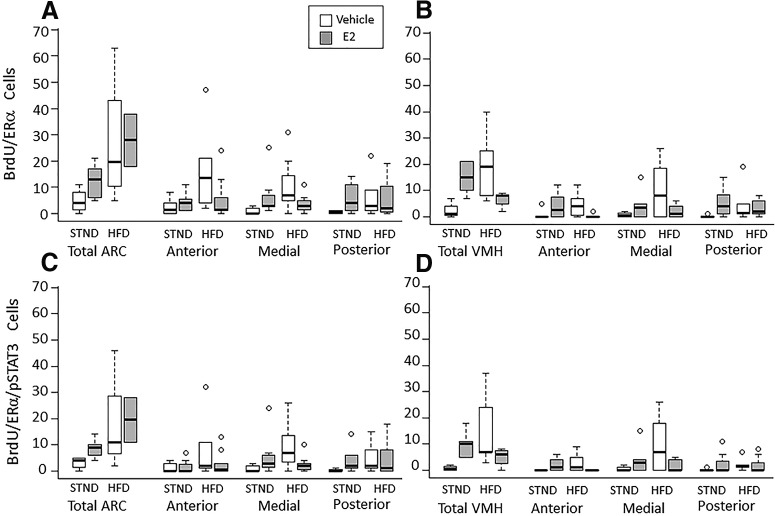
Estradiol and diet interact to regulate the number of newborn neurons that express ERα in the ARC and VMH of the adult female mouse. ***A***, ***B***, Double-labeled BrdU- and ERα-expressing cells were seen through the rostral–caudal extent of the ARC (***A***) and VMH (***B***). There was an interaction between hormone and diet with an effect on the number of newborn ERα-expressing cells in the medial region of both areas. ***C***, ***D***, In the ARC (***C***) and VMH (***D***), a subpopulation of the newborn estrogen-sensitive (ERα) and leptin-sensitive (pSTAT3) neurons was identified. There was an interaction between hormone and diet with an effect on the number of these triple-labeled cells in the medial regions of both the ARC and VMH. Interaction between hormone and diet, *p* < 0.05; *post hoc* analysis revealed no significant differences between groups (see text for further explanation).

Neurons triple labeled with BrdU, ERα, and pSTAT3 were observed in every region analyzed (Figs. 1, 3*C*,*D* ). There was a significant interaction between hormone and diet on the number of new neurons expressing both ERα and pSTAT3. This effect was seen in the medial ARC (*F*_(1,27)_ = 5.88, *p* = 0.023^q^), and the anterior (*F*_(1,26)_ = 7.09, *p* = 0.014^r^) and medial (*F*_(1,25)_ = 4.77, *p* = 0.04^s^) VMH. Although further analysis revealed no significant differences between groups, as was observed for new ERα cells above, there was a pattern for HFD consumption to increase the number of newborn ERα-pSTAT3-expressing cells in the medial ARC (*p* = 0.152^t^, STND-Veh vs HFD-Veh) and the medial VMH (*p* = 0.140^u^, STND-Veh vs HFD-Veh), and for E2 to attenuate this effect (*p* = 0.145^v^ for medial ARC; and *p* = 0.096^w^ for medial VMH; HFD-Veh vs HFD-E2).

### The number of ERα-expressing cells and leptin-sensitive ERα-expressing neurons is affected by diet

**Figure 4.**
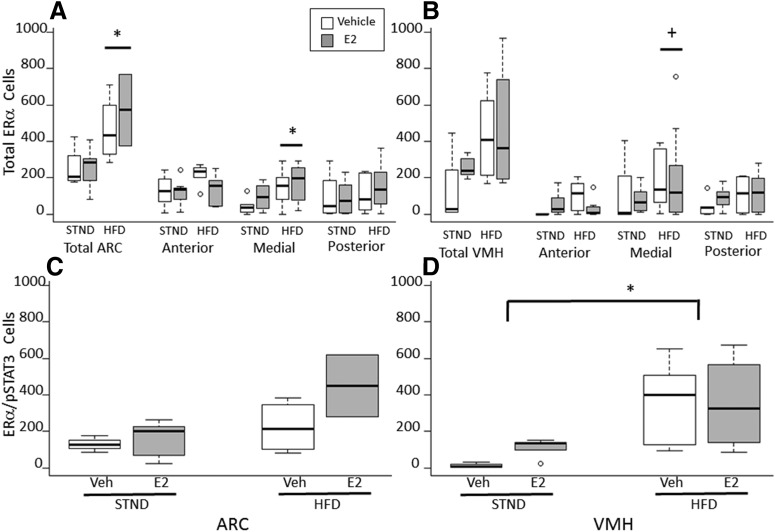
HFD increased the number of ERα-expressing cells. ***A***, HFD increased the number of ERα-expressing cells in the ARC, regardless of hormone treatment. This effect of HFD consumption was detected in the medial, but not anterior or posterior, ARC. ***B***, There was a strong trend towards an HFD-induced increase in ERα expression in the medial, but not anterior or posterior, VMH. ***C***, ***D***, HFD increased the number of leptin-sensitive ERα neurons in the VMH, but not in the ARC. *p < 0.05, +*p* = 0.054, HFD vs STND.

There was a main effect of diet on the number of ERα-expressing cells. The number of cells that contain ERα was greater in mice fed an HFD than those fed standard chow, regardless of hormone status (Fig. 4*A*,*B*). This effect of HFD was found in the medial ARC (*F*_(1,27)_ = 4.55, *p* = 0.043^x^), and a strong trend was found in the medial VMH (*F*_(1,25)_ = 4.29, *p* = 0.054^y^). In addition, HFD increased the number of leptin-sensitive ERα (ERα^+^/pSTAT3^+^) neurons in the total VMH (*F*_(1,17)_ = 4.697, *p* = 0.048^z^), but not in the ARC (Fig. 4*C*,*D*). There was no effect of estradiol treatment on ERα expression in the ARC or VMH. These results are consistent with previous findings that long-term estradiol treatment in female mice did not alter hypothalamic ERα expression (Temple et al., 2001).

### The density of ERα/pSTAT3-expressing neurons is greatest in the medial ARC

**Figure 5.**
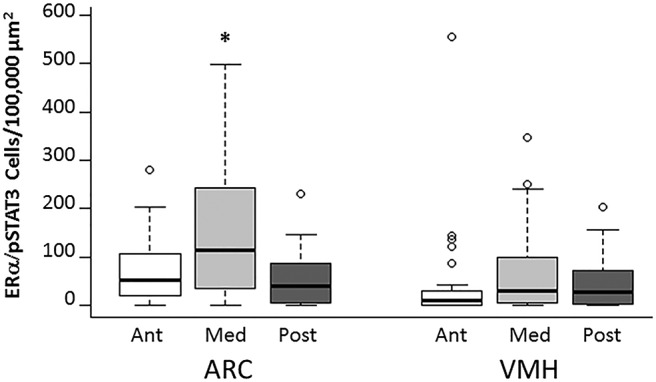
The medial ARC has the highest density of neurons that respond to both estrogens and leptin. The density of double-labeled ERα- and pSTAT3-positive neurons was greatest in the medial ARC compared with all other brain regions. *p < 0.05 compared with all other regions.

There was a main effect of ROI on the density (cells/100,000 µm^2^) of double-labeled ERα and pSTAT3 neurons across treatment groups (*F*_(5,154)_ = 5.302, *p* < 0.001^aa^; Fig. 5). *Post hoc* analysis found that the density of double-labeled neurons was greatest in the medial ARC region compared with all other regions (*p* = 0.014^bb^ for anterior ARC, *p* = 0.002^cc^ for posterior ARC, *p* < 0.001^dd^ for anterior VMH, *p* = 0.02^ee^ for medial VMH, *p* < 0.001^ff^ for posterior VMH).

### *Fgf10* gene expression is downregulated by E2 in mice on an HFD

RT-qPCR analysis of hypothalamic tissue from mice consuming an HFD found that estradiol treatment decreased *Fgf10* gene expression compared with Veh-treated mice (Table 2; *p* = 0.027^gg^). No differences were detected between hormone groups in *Bcl2*, *BDNF*, *capsase3*, *CNTF* or *IκB* gene expression. RNA integrity was verified by running all samples on a denaturing gel, resulting in a 28S/18S ratio of ≥2.

**Table 2:** Estradiol alters hypothalamic gene expression in female mice fed a HFD

Gene of Interest	Veh-HFD	E2-HFD
Bcl-2	10.56 (±0.74)	8.74 (±0.89)
BDNF	1.23 (±0.13)	0.77 (±0.24)
Caspase3	9.93 (±3.54)	16.15 (±4.37)
CNTF	1.80 (±0.54)	1.04 (±0.44)
Fgf10	1.13 (±0.43)	0.22 (±0.17)*
IκB kinase β	3.16 (±1.23)	3.45 (±1.23)

Estradiol decreased the relative expression of *Fgf10* in the hypothalamus of female mice fed a HFD.

* *p* = 0.027^gg^ E2 vs Veh.

## Discussion

### The adult female mouse brain generates new ERα cells

Along with the hippocampus and olfactory bulb, it is now well accepted that the adult mammalian hypothalamus is capable of generating new neurons (Kokoeva et al., 2007). The phenotype of these newly generated hypothalamic neurons is just beginning to be elucidated in male mice (Pierce and Xu, 2010; Lee et al., 2012; McNay et al., 2012; Gouaze et al., 2013; Haan et al., 2013; Nascimento et al., 2016) and female mice (Bless et al., 2014). The current study shows that the adult female mouse brain produces new cells that express ERα, a receptor involved in reproduction (Ogawa et al., 1998; Emmen and Korach, 2003) and energy homeostasis (Heine et al., 2000; Santollo et al., 2007, 2010). Furthermore, the number of these new ERα-expressing cells is dependent on E2 level and diet. The addition of newborn ERα-expressing cells could function to enhance estrogen responsiveness and to provide a protective mechanism against obesity by increasing activity levels and decreasing feeding behavior (Wade, 1972; Blaustein et al., 1976; Xu et al., 2011). In addition, these newborn ERα cells could act to modulate reproductive behavior (Musatov et al., 2006; Gao and Horvath, 2008). Studies in adult male mice have found that some new hypothalamic neurons express peptides related to energy homeostasis, such as NPY (Kokoeva et al., 2005; McNay et al., 2012; Gouaze et al., 2013) and POMC (Kokoeva et al., 2005; McNay et al., 2012; Gouaze et al., 2013). Since E2 alters the expression of both NPY and POMC (Pelletier et al., 2007; Silva et al., 2010), it will be important for future studies to investigate whether the adult female mammalian brain also generates new neurons that express these feeding-related peptides and ERα.

### HFD increases the number of ERα-expressing neurons

While there is evidence that some glial cells are leptin responsive (Kim et al., 2014a), we found that virtually all leptin-sensitive cells in the medial ARC and VMH also expressed the neuronal marker Hu, indicating these hypothalamic pSTAT3 cells are neurons, and suggesting that the majority of these cells in the anterior and posterior ARC and VMH are neurons. Furthermore, many of the new hypothalamic pSTAT3 neurons identified here also express ERα, suggesting that these new neurons respond to estrogens and leptins. However, it should be noted that ERα has been identified in glial cells (Langub and Watson, 1992; Pawlak et al., 2005), which have been implicated in energy homeostasis (Argente-Arizón et al., 2015), suggesting that ERα can influence energy homeostasis via non-neuronal mechanisms. In addition, while it is well established that leptin induces the phosphorylation of STAT3 in the hypothalamus (Hübschle et al., 2001; Gao and Horvath, 2008), there are reports that estradiol injections can rapidly increase pSTAT3 levels in the hypothalamus within 30 min (Gao et al., 2007). Therefore, while it is possible that the slow-acting estradiol implants used in the present study induced pSTAT3, suggesting that not all hypothalamic pSTAT3-labeled cells in the present study are leptin sensitive, it is likely that the majority of the pSTAT3-labeled cells detected after leptin injection are leptin responsive. Finally, a recent study (Kim et al., 2016) suggests that the anorectic effects of estradiol may occur independently of pSTAT3 signaling pathways in the hypothalamus. It will be important for future studies to address the functional significance in energy homeostasis and reproduction of these new hypothalamic ERα-expressing neurons that respond to leptins.

The current study found an increase in ERα expression in the medial aspects of both the ARC and VMH in mice fed an HFD compared with those fed standard chow, regardless of hormonal status. In addition, mice consuming an HFD had a higher number of leptin-sensitive ERα-expressing neurons compared with those fed standard chow. This effect was seen in the VMH, but not in the ARC. In further support of a role for ERα in the VMH in energy homeostasis, RNAi to ERα in this region resulted in a decreased response to E2 in weight loss and adiposity (Musatov et al., 2007). Consistent with the present findings that consuming an HFD elevates hypothalamic ERα levels, consuming an HFD increased ERα expression in the hypothalamus of prepubescent female pigs (Zhuo et al., 2014) and mouse mammary gland (Hilakivi-Clarke et al., 1998). However, long-term (16 weeks) consumption of an HFD in mice decreased hypothalamic ERα expression in males, but not in intact females, suggesting a sex-specific effect (Morselli et al., 2014). E2 acts through ERα to decrease hypothalamic inflammation (Morselli et al., 2014), and decreased inflammation is correlated with weight maintenance (Yu et al., 2013). Together with the present findings, these studies suggest that HFD consumption triggers increases in hypothalamic ERα and downstream signaling molecules (e.g., pSTAT3) as a protective mechanism to maintain homeostasis by providing an increase in the components of the estrogenic–anorectic pathway.

### Estrogens and leptin interact in the VMH and ARC

While coexpression of leptin receptors and ER has been localized to areas of the hypothalamus (Diano et al., 1998), the precise region of the greatest estrogen-pSTAT3 sensitivity in the hypothalamus has not yet been elucidated. In the present study of the anterior, medial, and posterior ARC and VMH, the greatest density of neurons coexpressing ERα and pSTAT3 was in the medial ARC, suggesting strong interaction between the estrogen and leptin signaling pathways in the medial ARC. In support, E2 microinjections directly into the ARC decrease food intake in ovariectomized rats (Santollo et al., 2011). In addition, all ARC cells containing ERα also express leptin receptor in female rats (Diano et al., 1998).

### *Fgf10* gene expression is associated with an increase in neurogenesis

We tested the hypothesis that estradiol regulates hypothalamic growth factors or cytokines known to be involved in neurogenesis (Endres et al., 1998; Fink et al., 1998; Pencea et al., 2001; Kokoeva et al., 2005; Sasaki et al., 2006; Zhang et al., 2006; Yuan, 2008; Zhao et al., 2008; Choi et al., 2009; Li et al., 2012). Analysis by RT-qPCR revealed that *Fgf10* gene expression was greater in the hypothalamus of Veh-treated mice, indicating that estradiol treatment decreased *Fgf10* gene expression. While the hypothalamic neurogenic niche has not been identified, studies have suggested that there are β-tanycytes with stem cell potential along the ventral third ventricle (Lee et al., 2012; Bolborea and Dale, 2013). Moreover, these tanycytes express Fgf10 and may be the precursors for the generation of new cells in the hypothalamus (Haan et al., 2013). Together with previous studies, the present findings suggest that endogenous estradiol downregulation of Fgf10 may play a role in cell proliferation and neurogenesis in the hypothalamus of HFD-fed animals. No changes were detected in the gene expression of other factors previously shown to have a role in cell proliferation, survival, or apoptosis (CNTF, BCl-2, caspase 3, BDNF, and IKKβ; Endres et al., 1998; Pencea et al., 2001; Kokoeva et al., 2005; Sasaki et al., 2006; Li et al., 2012). Given that the whole hypothalamus was analyzed in the present study, it will be important for future experiments to use higher neuroanatomical resolution when exploring the role of these factors in neurogenesis in the ARC and VMH.

In summary, new cells were identified in the adult female hypothalamus that express ERα. Furthermore, a subpopulation of these new ERα cells also express pSTAT3, indicating that they are neurons. Interestingly, the birth of these new hypothalamic estrogen- and leptin-responsive neurons is influenced by diet and hormonal condition. These findings suggest a novel mechanism by which E2 can affect energy homeostasis in females.

## Synthesis

The decision was a result of the Reviewing Editor Jeffrey Blaustein and the peer reviewers coming together and discussing their recommendations until a consensus was reached. A fact-based synthesis statement explaining their decision and outlining what is needed to prepare a revision is listed below. The following reviewer(s) agreed to reveal their identity: Toni Pak

The reviewers and editor agree that there a good deal of merit to this manuscript. However, there are also many problems that need to be dealt with before it would be acceptable for publication in eNEURO. It will therefore be necessary to tend to all of the comments of the reviewers before resubmitting for further consideration.

Reviewer 1

The current study investigates the role of estrogen (E2) and high fat diet (HFD) on the generation of new neurons expressing estrogen receptor alpha (ERalpha) in the adult female hypothalamus. For experiment 1, female adult mice were ovariectomized and treated with oil (Veh) or E2, then provided a standard chow diet or a HFD. The authors found a significant interaction of hormone and diet on the generation of new cells expressing ERalpha. In the standard diet, female mice treated with E2 showed an increase in BrdU/ERalpha-labeled cells compared to Veh mice. However, in the HFD, the opposite effect was found, with E2 decreasing the number of BrdU/ERalpha-labeled cells in E2-treated females compared to Veh. The same interaction was also found in BrdU/ERalpha/pSTAT3 cell counts. In experiment 2, the authors examined the effects of E2 on protein expression (Fgf10, CNTF, BDNF, Bcl2, etc.) when adult female mice were given a HFD. They found that Fgf10 protein expression was decreased in E2-treated females compared to Veh. Thus, the decrease in ERalpha neurogenesis seen in the E2-HFD mice compared to Veh-HFD mice may be due to Fgf10. The data from this study are novel and provide important insight regarding how estrogen can affect the brain differently in response to diet. However, the manuscript itself leaves much to be desired. The authors use brain tissue from a previous study, which is great as it reduces number of animals used, but important information from the previous published study is left out of the current manuscript (i.e. food intake, weight gain, locomotion, etc.), which makes it difficult to interpret the results in the current manuscript. The authors discuss some information from the previous study (i.e. total number of BrdU cells), but they should include more to create a more complete story. Additional comments for improvement/clarification are listed below:

1. How many animals were used in these studies-what were sample sizes for the various groups in each experiment? More details on the IHC analyses are needed. How many brain sections (and how far apart) per ROI per animal were used for these analyses?

2. Authors need to include the required statistical table that shows the statistical power of each analysis completed in this study. It would also be helpful for author to include the actual p-values, means, and SE-values in a table or within the text itself.

3. The authors only include data from significant ROIs. They should also include non-significant data from the other ROIs (i.e. anterior/posterior ARC) analyzed (means, SE, p-values).

4. What are the DV coordinates for the BrdU cannulas?

5. The authors should include photomicrographs for the BrdU/Hu/pSTAT3 images. If virtually all the pSTAT3 cells expressed Hu, it would be nice to have visual confirmation. Also, as these are new data, they should include all data used to complete the statistical analyses. For example, in lines283-287, authors should report the total number of pSTAT3 cells examined in ARC and VMH, not just the proportion of cells that expressed Hu.

6. Authors should temper their claim that all pSTAT3-labeled cells are leptin-responsive. There is evidence, which the authors cite, that estrogen by itself can cause STAT3 phosphorylation.

7. The authors report a hormone by diet interaction for the number of new ERalpha neurons and ERalpha/pSTAT3 neurons, but these results are not clearly described or discussed. Lines 275-276 state that HFD increases BrdU/ERalpha neurons and that E2 attenuates this effect, but to my eye, the interaction is that E2 has different effects depending on diet (increases new cells in standard diet, decreases new cells in HFD). As far as I can tell, the interaction was not probed statistically, and if this were done, that might help clarify the nature of the interaction.

8. Related to point 7, the interaction referred to in Lines 289-90 should be described; it's not sufficient just to say there's an interaction. Figure 2 is little help, as the significance bars/asterisks are not informative. In fact they are somewhat misleading because they seem to indicate a main effect, not the interaction as indicated in the caption. What is the exact nature of the interaction, and were follow up statistics performed to probe the interaction?

9. In line 402-403, the authors state, "…fgf10 [is] a possible mechanism by which HFD increases hypothalamic neurogenesis in the absence of E2." In order to make this claim, the authors need to examine Fgf10 gene expression in standard chow group as well. With the current results, the authors can only really infer that estrogen reduces Fgf10 expression, but cannot attribute this finding to high fat diet.

10. Authors should be careful in claims on line 340-342. Not all pSTAT3 cells in the ARC and VMH expressed Hu. The authors' data only show that pSTAT3 cells are neurons in the medial portions of these brain regions.

11. For me, the most interesting result is that estrogen has a different effect on neurogenesis of ERalpha-expressing cells, depending on diet. Authors do not provide a possible rationale for this finding, nor do they actually address what these new ERalpha neurons could be doing in terms of behavior. What might be the purpose for these ERalpha/leptin responsive neurons?

Reviewer 2

The studies described in this manuscript test the hypothesis that high fat diet and estradiol (E2) contribute to the regulation of neurogenesis in the arcuate and ventral medial hypothalamus of adult female mice. There are some potentially interesting findings, however there is a lack of clear methodological details and some confusing results that make the merits of the study difficult to evaluate. The following suggestions are designed to help improve the readability of the manuscript and understanding for the significance of the study.

Methods -

1. It is not clear how many total mice were used for these studies, the N/group, and whether or not any were excluded (if yes, what were the exclusion criteria). Given the reported F-values it would appear that there were some variations in N between groups (e.g. line 299: F (df2) = 27, but line 301, F (df2) = 17).

Minor:

2. PCR - line 230 "(company information)" was listed after nanodrop. Also, Nanodrop values do not indicate RNA integrity - were agarose gels also run on each sample to check quality?

3. PCR - current nomenclature guidelines suggest using RT-qPCR as the abbreviation to denote reverse transcription-quantitative PCR.

Results -

1. First paragraph - The purpose of including this paragraph in the results section is not clear. It would seem that most of the paragraph is referring to a previously published study. Contrasts/similarities between the current data and previous studies should be restricted to the discussion section.

2. Line 305 - Why is the F (df1) value so high (= 5)? The methods described the design as a 2 x 2 (diet and hormone), so the (df1) should equal 1. The associated bar graph (Fig. 4) does not clear things up, because it is not specified if these animals were treated with E2 or had different diets. Also, the F (df2) is very high (=154) and inconsistent with the (df2) in the other experiments. Again, reporting the N/group for each experiment would be helpful.

3. Some statistically significant interactions were reported, but no mention of post-hoc between group differences. A bar with asterisk to denote interactions is unusual and potentially misleading.

4. Figure 1 - what was the treatment for this mouse? Also, scale bars should be included on each figure.

5. Plasma estradiol levels should be reported in order to gain an idea of which part of the estrous cycle these data would reflect.

6. The bar graphs have very high SEM. It would be helpful if these data were reported as a dot box plot instead, as the bar graph makes it difficult to know whether there were a few outliers, or if there was overall high variability among all the animals in the group.

Discussion -

The discussion lacks some depth of analysis for the data reported. For instance, why would E2 not have any effect on the number of ERalpha+ cells (Fig. 3)? Typically, chronic levels of E2 (as would be achieved with a silastic capsule) is thought to downregulate the number of ERalpha expressing cells in the hypothalamus. (Mahavongtrakul et al., 2013, Endo; Bondar et al., 2009 J. Neuroendo; Brown TJ et al., 1996 Neuroendo).

The authors report that HFD alone, regardless of hormone status, increases ERalpha+ cells and there is no effect of hormone treatment. Yet, there was an effect of hormone treatment on new cells (decrease), suggesting that E2 might actually increase ERalpha expression in existing cells, compared to vehicle, resulting in what would appear to be a net effect of no change. On the other hand, the numbers of new cells were very few and the error bars high, so a difference might not have been detected. Some comments on this would be useful.

The Fgf10 data are premature to make any conclusions regarding mechanism, especially since it was assayed from whole hypothalamus and not the regions where neurogenesis was measured. Also, it isn't clear why the other markers were chosen to measure, and what the significance is for the lack of observed effects. Some discussion on this point should be added.
